# Assessing the Quality and Safety of Do‐It‐Yourself Cosmetic Neuromodulator Injection Tutorials on YouTube

**DOI:** 10.1111/jocd.70252

**Published:** 2025-05-20

**Authors:** Lauren Ching, Christopher Guirguis, Andrew Elliott, Amir A. Hakimi, Michael J. Reilly

**Affiliations:** ^1^ Georgetown University School of Medicine Washington, DC USA; ^2^ Ohio State University Columbus Ohio USA; ^3^ Department of Otolaryngology—Head and Neck Surgery MedStar Georgetown University Hospital Washington, DC USA

**Keywords:** botulinum toxin, intradermal injection, patient education

To the editor,

The surge in demand for Botulinum toxin injection has sparked a trend of patients self‐administering Botulinum Toxin A as well as other unregulated neuromodulators purchased through direct‐to‐consumer online retailers [1]. It is important for consumers and practitioners to recognize the risks associated with these do‐it‐yourself (DIY) practices, particularly when performed by untrained individuals.

Our group previously demonstrated that YouTube includes potentially unsafe DIY cosmetic filler injection tutorials [2]. To our knowledge, a similar study assessing the quality of DIY cosmetic neuromodulator injection tutorials has yet to be performed. Herein, we sought to evaluate the quality and safety of DIY neuromodulator injection videos on YouTube based on their discussion and/or demonstration of safe injecting techniques as described by the Allergan Aesthetics' Medication Guide Insert (MGI) [3].

YouTube was queried on April 27, 2025, using the search term “DIY Botox”. The first 101 videos were screened and included if they were in English and either demonstrated injection techniques on a mannequin or human or gave instructions on injection using injection site maps. The first 101 videos were reviewed as it would be unlikely for viewers to watch more videos in a single search. The seven guidelines that formed the basis for assessing content quality and adherence were derived from the MGI from Allergan Aesthetics [3]. Videos were evaluated for guideline adherence, injector credentials, discussion of safety measures, and viewer engagement metrics.

Among the 101 videos assessed, 40 (40%) met inclusion criteria. These videos had an average of 192 618 views (SD = 199 722) and 3784 likes (SD = 6918). Videos covered an average of 1.51 (SD = 1.22) out of the seven guidelines. A breakdown of guidelines adherence can be found in Table 1. Most videos covered treatment of the frontalis (65%) and glabellar regions (59%). Most videos demonstrated injections on humans/mannequins (89%) and used sterile technique (82%). Approximately 39% of the videos lacked a discussion of complications and when to seek medical attention. Few videos included a demonstration of sharps disposal (4.7%) or a safety disclaimer within the video or description (31%). Most injectors did not state their credentials in the video (73%), while 15% were physicians and 12% were nurses. 81% of the videos demonstrated the use of Botox, while the remaining videos used Dysport (8.5%), ReNTox (1.3%), and Nabota (1.3%); the remaining 7.9% of videos did not identify the compound being used.

**TABLE 1:** Summary of guideline adherence across assessed videos.

Guideline	Video adherence (%)
Reconstitution and dilution process	7.5%
Understanding of neuromuscular and structural anatomy	66%
Proper dose determination based on muscle activity	17%
Frequency of administration for safety and effectiveness	18%
Identification of injection sites and anatomical landmarks	20%
Safe and effective use including storage and time frames post‐reconstitution	4.4%

The findings highlight a significant gap in the quality and reliability of DIY neuromodulator tutorials on YouTube. Wong et al. previously assessed the quality of Botulinum toxin YouTube videos as educational resources for patients, finding most videos to provide helpful patient information [4]. Although general information regarding cosmetic neuromodulator injection may serve as an educational resource, our study highlights that the techniques demonstrated in many YouTube videos were largely unsafe and/or insufficient. Some videos describe the use of neuromodulators that are not approved by the United States Food and Drug Administration. Despite high viewer engagement, the lack of guideline adherence and professional credibility among video creators underscores the potential risks of misinformation and complications among unsuspecting consumers.

The popularity of neuromodulator injection tutorials, juxtaposed with the scarcity of credible and educational content, echoes the concerns on the reliability of health information on YouTube [5, 6]. These studies emphasize the need for viewers to critically assess online health content and highlight the role of policy makers in regulating online health information.

This study underscores the need for caution among viewers seeking information on neuromodulator injections on YouTube. Healthcare professionals should play a proactive role in educating patients, offering standardized and accurate information to counter the incomplete and/or misinformation found on platforms like YouTube.

## Author Contributions

L.C., C.G., A.E., A.A.H. performed data collection. L.C., C.G., A.A.H. performed data analysis. L.C., C.G., A.E. wrote the paper. A.A.H., M.J.R. provided critical edits to the paper. A.A.H., M.J.R. designed the research study.

## Conflicts of Interest

The authors declare no conflicts of interest.

## Data Availability

The data that support the findings of this study are available from the corresponding author upon reasonable request.
